# Carboxyl Group-Modified Myoglobin Induces TNF-α-Mediated Apoptosis in Leukemia Cells

**DOI:** 10.3390/ph15091066

**Published:** 2022-08-27

**Authors:** Yuan-Chin Lee, Jing-Ting Chiou, Liang-Jun Wang, Yi-Jun Shi, Ying-Jung Chen, Long-Sen Chang

**Affiliations:** 1 Institute of Biomedical Sciences, National Sun Yat-sen University, Kaohsiung 804, Taiwan; 2 Department of Fragrance and Cosmetic Science, Kaohsiung Medical University, Kaohsiung 807, Taiwan; 3 Department of Biotechnology, Kaohsiung Medical University, Kaohsiung 807, Taiwan

**Keywords:** myoglobin, modification of carboxyl group, NOX4/SIRT3/p38 MAPK axis, tristetraprolin suppression, TNF-α-mediated death pathway

## Abstract

Previous studies have shown that chemical modification may increase the activity of proteins or confer novel activity to proteins. Some studies have indicated that myoglobin (Mb) is cytotoxic; however, the underlying mechanisms remain unclear. In this study, we investigated whether chemical modification of the carboxyl group by semicarbazide could promote the Mb cytotoxicity in human leukemia U937 cells and the underlying mechanism of semicarbazide-modified myoglobin (SEM-Mb)-induced U937 cell death. The semicarbazide-modified Mb (SEM-Mb) induced U937 cell apoptosis via the production of cleaved caspase-8 and t-Bid, while silencing of FADD abolished this effect. These findings suggest that SEM-Mb can induce U937 cell death by activating the death receptor-mediated pathway. The SEM-Mb inhibited miR-99a expression, leading to increased NOX4 mRNA and protein expression, which promoted SIRT3 degradation, and, in turn, induced ROS-mediated p38 MAPK phosphorylation. Activated p38 MAPK stimulated miR-29a-dependent tristetraprolin (TTP) mRNA decay. Downregulation of TTP slowed TNF-α mRNA turnover, thereby increasing TNF-α protein expression. The SEM-Mb-induced decrease in cell viability and TNF-α upregulation were alleviated by abrogating the NOX4/SIRT3/ROS/p38 MAPK axis or ectopic expression of TTP. Taken together, our results demonstrated that the NOX4/SIRT3/p38 MAPK/TTP axis induces TNF-α-mediated apoptosis in U937 cells following SEM-Mb treatment. A pathway regulating p38 MAPK-mediated TNF-α expression also explains the cytotoxicity of SEM-Mb in the human leukemia cell lines HL-60, THP-1, K562, Jurkat, and ABT-199-resistant U937. Furthermore, these findings suggest that the carboxyl group-modified Mb is a potential structural template for the generation of tumoricidal proteins.

## 1. Introduction

Chemical modifications have been widely used to explore the functional significance of certain amino acid residues in proteins. If modifications of proteins result in a decrease in their biological activities, it can be inferred that these modified residues are important for the manifestation of protein activity [1,2,3]. However, experimental evidence has indicated that the activity of *Candida antarctica* and *Thermomyces lanuginose* lipase increases after modification of the carboxyl group using ethylenediamine [4]. Modification of Lys and Arg residues with maleic anhydride and 3-hydroxyphthalic anhydride confers anti-HIV activity to ovalbumin, which lacks antiviral activity prior to chemical modification [5,6]. Glycated bovine serum albumin and ovalbumin with carboxyl groups modified with *p*-aminophenyl α-D-mannopyranoside show membrane-permeabilizing and antibacterial activities [7,8,9,10]. The use of semicarbazide to block the negative charge in the carboxylic acid groups of α-lactalbumin (LA) enables it to destabilize bilayer membranes and induce cancer cell death [11,12]. These results indicate that chemical modifications can increase the protein activity and even create novel functions. Notably, semicarbazide-modified LA exhibits characteristics of a molten globule state [11]. In contrast, the lipid-protein complex formed by Ca^2+^-free apo-α-lactalbumin (apo-LA) and oleic acid exhibits tumoricidal activity associated with the molten globule-like conformation of apo-LA [13,14]. These findings suggest that chemical modifications may unlock intrinsically active regions masked in the protein structure by altering protein conformation, allowing modified protein to exhibit novel activity.

Myoglobin (Mb) is a monomeric O_2_-binding heme protein typically present in the cardiac and aerobic skeletal muscles of vertebrates and is responsible for the storage and transport of O_2_ [15]. The α-helix is the dominant secondary structure in Mb and apomyoglobin (apo-Mb) [16]. Selective cleavage of apo-Mb at Met55 and Met131 by CNBr yields the apo-Mb 56-131 peptide, causing a loss of α-helix [17]. In contrast to apo-Mb and Mb, the apo-Mb 56-131 peptide exhibits membrane leakage-inducing activity in physiological solutions [17]. These findings imply that the intact structure of Mb and apo-Mb hinders the active region at positions 56-131, exhibiting membrane-permeabilizing activity. This suggests that the structural changes in apo-Mb or Mb potentially expose the active region inside the proteins. Growing evidence indicates that Mb is expressed in breast cancer, prostate cancer, squamous cell carcinoma of the head and neck, non-small cell lung cancer, renal cell cancer, and colon cancer [18,19,20,21,22]. Breast cancer patients with higher Mb expression in tumor tissues have a better prognosis [23]. In contrast, treatment with 10 mg/mL Mb resulted in an approximately 60% to 85% reduction in the viability of the human proximal tubular cell line HK-2 [24]; however, the mechanism of Mb cytotoxicity is largely unknown. Based on these findings, we speculated that the anti-cancer activity of Mb is hindered by its structure, and that distortion of the Mb structure may increase its tumoricidal effect. As modification of the carboxyl group in LA with semicarbazide simultaneously changed its conformation and conferred the protein with anti-cancer effects [8], we envisioned the possibility of distorting the α-helical structure of Mb through carboxyl group modification to generate anti-cancer activity. Recent studies have shown that modification of the carboxyl group in Mb with semicarbazide profoundly distorts its gross structure [25]. Therefore, in this study, we investigated the cytotoxicity of semicarbazide-modified Mb (SEM-Mb). A previous study has confirmed that chemical modification of the carboxyl group enables LA to display cytotoxicity in human acute myeloid leukemia (AML) U937 cells [8]. Therefore, we used U937 cells to analyze the anti-leukemic activity and mechanism of SEM-Mb. 

## 2. Results

### 2.1. The Death Receptor-Mediated Apoptotic Pathway Is Involved in SEM-Mb-Mediated Cytotoxicity

**Figure 1:**
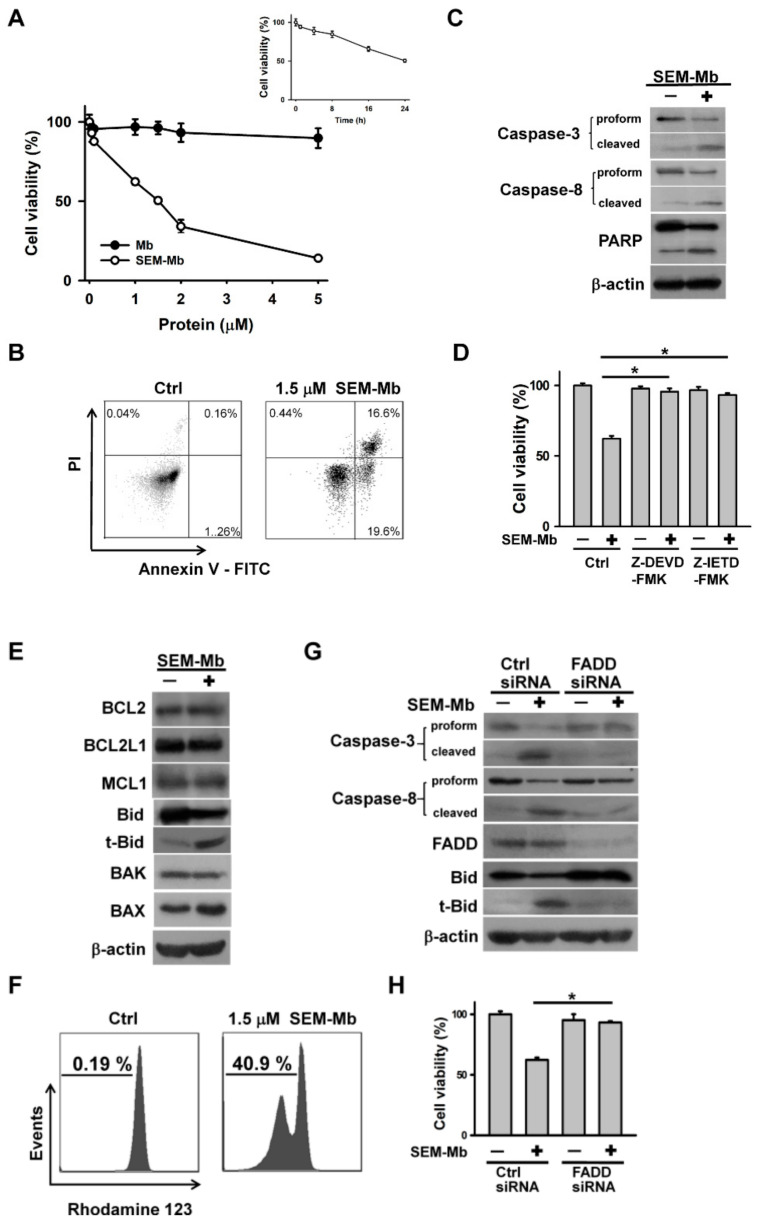
SEM-Mb-treated U937 cells underwent apoptosis. Without specific indication, U937 cells were treated with 1.5 μM SEM-Mb for 24 h. (**A**) SEM-Mb induced cell death in a concentration- and time-dependent manner. U937 cells were incubated with varying concentrations of SEM-Mb or Mb for 24 h. Cell viability was determined by MTT assay. Results are expressed as the percentage of cell survival relative to the control. Each value is the mean ± SD of triplicate determinations. (Inset) U937 cells were incubated with 1.5 μM SEM-Mb for indicated time periods. (**B**) Flow cytometry analyses of SEM-Mb-treated U937 cells using annexin V-FITC/propidium iodide double staining. (Left) Untreated control cells. (Right) U937 cells were treated with 1.5 μM SEM-Mb for 24 h. On flow cytometric scatter graphs, the left lower quadrant represents remaining live cells. The right lower quadrant represents the population of early apoptotic cells. The right upper quadrant represents the accumulation of late apoptotic cells. (**C**) Western blot analyses showing the degradation of procaspase-3/-8 and PARP in SEM-Mb-treated cells. (**D**) Pretreatment with caspase inhibitors restored the viability of SEM-Mb -treated cells. U937 cells were pretreated with 10 μM Z-DEVD-FMK (caspase-3 inhibitor) or Z-IETD-FMK (caspase-8 inhibitor) for 1 h, and then incubated with 1.5 μM SEM-Mb for 24 h. Cell viability was determined by MTT assay. The values represent averages of three independent experiments with triplicate measurement (mean ± SD, * *p* < 0.05). (**E**) Effect of SEM-Mb on the production of t-Bid and the expression of BCL2 family proteins. (**F**) Dissipation of mitochondrial membrane potential (∆Ψm) in SEM-Mb-treated cells. U937 cells were treated with 1.5 μM SEM-Mb for 24 h, and then incubated with rhodamine 123 for 15 min. The loss of ∆Ψm was analyzed by flow cytometry. (**G**) Transfection of FADD siRNA inhibited SEM-Mb-induced the degradation of procaspase-8/-3 and the production of t-Bid. U937 cells were transfected with 100 nM control siRNA or FADD siRNA, respectively. After 24 h post-transfection, the cells were treated with 1.5 μM SEM-Mb for 24 h. (**H**) Depletion of FADD inhibited SEM-Mb-induced cell death (mean ± SD, * *p* < 0.05).

Unlike Mb, SEM-Mb was cytotoxic to the U937 cells (Figure 1A). The 50% inhibitory concentration (IC50) was approximately 1.5 μM after 24 h of treatment (Figure 1A). SEM-Mb-mediated cytotoxicity was further investigated using a single dose of 1.5 μM. The SEM-Mb treatment increased the number of annexin V-stained cells (Figure 1B) and induced degradation of procaspase-3/-8 and PARP (Figure 1C). Inhibition of caspase-3/-8 activity maintained cell viability even after SEM-Mb treatment (Figure 1D), suggesting that SEM-Mb-treated U937 cells underwent apoptosis. There is compelling evidence that t-Bid, produced by caspase-8-mediated cleavage of Bid, triggers disruption of the mitochondrial membrane potential (∆Ψm), leading to mitochondria-mediated apoptosis [26,27]. The SEM-Mb-treated U937 cells produced t-Bid (Figure 1E) and caused mitochondrial membrane depolarization (Figure 1F), suggesting that SEM-Mb-induced apoptosis is related to the activation of the caspase-8/mitochondria-mediated pathway. Treatment of U937 cells with SEM-Mb did not alter the expression of MCL1, BCL1, BCL2L1, and BAK but increased the expression of BAX protein. Accumulating evidence indicates that the TNF-family death receptors recruit FADD and procaspase-8 to activate caspase-8, thereby triggering caspase-3-mediated apoptosis [27]. Knockdown of FADD attenuated the cleavage of caspases-8/-3 and Bid in the cells exposed to SEM-Mb (Figure 1G). Furthermore, FADD silencing reduced SEM-Mb-induced cell death (Figure 1H). These findings indicated that the death receptor-mediated apoptotic pathway is involved in SEM-Mb-mediated cytotoxicity.

### 2.2. SEM-Mb Induces Ca^2+^-Elicited ROS Generation, Which in Turn Reduces U937 Cell Viability

In a previous study, Zager and Burkhart [24] reported that Mb-induced HK-2 cell death is related to Ca^2+^ and ROS signaling pathways. Thus, we analyzed whether Ca^2+^ and ROS were involved in the cytotoxicity of SEM-Mb against U937 cells. The SEM-Mb treatment triggered maximal ROS production after 6 h of treatment (Supplementary Figure S1A) and continued to increase the intracellular Ca^2+^ concentration ([Ca^2+^]i) over 24 h (Supplementary Figure S1B). The BAPTA-AM (a Ca^2+^ chelator) pretreatment alleviated ROS production induced by SEM-Mb (Supplementary Figure S1C), whereas NAC (an ROS scavenger) did not reduce [Ca^2+^]i in SEM-Mb-treated cells (Supplementary Figure S1C). Intracellular ROS are mainly produced by mitochondria and NADPH oxidase (NOX) [28,29]. Therefore, we analyzed the effect of NOX inhibitors on SEM-Mb-induced ROS production. Pretreatment with BAPTA-AM, NAC, and GLX351322 (an NOX4 inhibitor), but not ML171 (an NOX1 inhibitor), reduced ROS generation in SEM-Mb-treated cells (Supplementary Figure S1D). The GLX351322 failed to inhibit the SEM-Mb-induced [Ca^2+^]i elevation (Supplementary Figure S1C). SEM-Mb treatment also increased mitochondrial ROS levels (Supplementary Figure S1E). The BAPTA-AM, NAC, GLX351322, and Mito-TEMPO (mitochondria-targeted antioxidant) attenuated the effects of SEM-Mb on mitochondrial ROS production, ∆Ψm loss, and cell death (Supplementary Figure S1F–H). Evidently, SEM-Mb induced Ca^2+^-elicited ROS generation, which, in turn, reduced U937 cell viability.

### 2.3. SEM-Mb Induces Ca^2+^-Mediated Downregulation of miR-99a, Leading to Upregulation of NOX4 Expression

**Figure 2:**
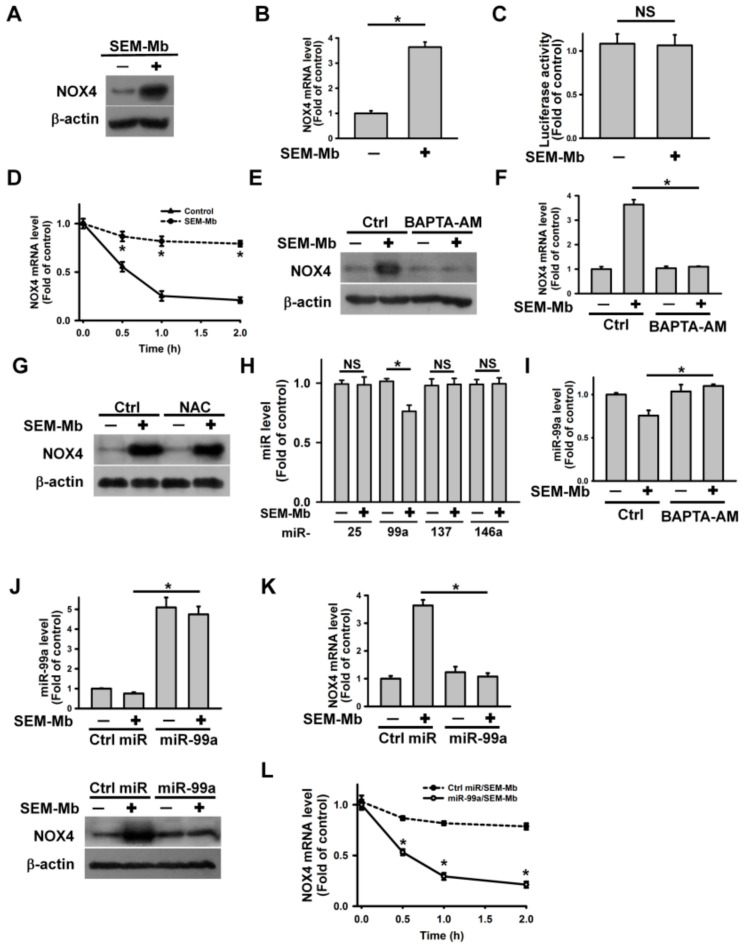
SEM-Mb treatment increased NOX4 expression in U937 cells. U937 cells were directly treated with 1.5 μM SEM-Mb for 24 h or pre-treated with 10 μM BAPTA-AM or 2 mM NAC for 1 h and then incubated with 1.5 μM SEM-Mb for 24 h. (**A**) SEM-Mb induced NOX4 upregulation. (**B**) Detecting the transcription of NOX4 mRNA using qRT-PCR. The values represent averages of three independent experiments with triplicate measurements (mean ± SD, * *p* < 0.05). (**C**) Effect of SEM-Mb on the luciferase activity of NOX4 promoter. After transfection with pGL3-NOX4 for 24 h, the transfected cells were treated with 1.5 μM SEM-Mb for 24 h and then harvested for measuring luciferase activity. The data represent averages of three independent experiments with triplicate measurements (mean ± SD, NS, not statistically significant). (**D**) Effect of SEM-Mb treatment on NOX4 mRNA stability. Cells were treated with or without 1.5 μM SEM-Mb for 24 h, and then incubated with 10 μg/mL actinomycin D for the indicated time periods. The level of NOX4 mRNA was analyzed by qRT-PCR (mean ± SD, * *p* < 0.05). SEM-Mb-untreated and -treated cells without actinomycin D treatment were used as control. (**E**) Effect of BAPTA-AM on NOX4 protein expression in SEM-Mb-treated cells. (**F**) Effect of BAPTA-AM on NOX4 mRNA expression in SEM-Mb-treated cells (mean ± SD, * *p* < 0.05). (**G**) Effect of NAC on NOX4 protein expression in SEM-Mb-treated cells. (**H**) Effect of SEM-Mb on the expression of miR-25, miR-137, miR-146a, and miR-99a (mean ± SD, * *p* < 0.05; NS, not statistically significant). (**I**) Effect of BAPTA-AM on the expression of miR-99a in SEM-Mb-treated cells (mean ± SD, * *p* < 0.05). (**J**) Transfection of hsa-miR-99a inhibited SEM-Mb-induced NOX4 upregulation. Cells were transfected with negative control miRNA or hsa-miR-99a. After 24 h post-transfection, the cells were treated with 1.5 μM SEM-Mb for 24 h. (Top panel) qRT-PCR analysis of miR-99a levels in hsa-miR-99a-transfected cells (mean ± SD, * *p* < 0.05). (Bottom panel) Western blot analysis of NOX4 expression in hsa-miR-99a-transfected cells after SEM-Mb treatment. (**K)** Transfection of hsa-miR-99a inhibited SEM-Mb-induced increase in NOX4 mRNA expression (mean ± SD, * *p* < 0.05). (**L**) Transfection of hsa-miR-99a increased NOX4 mRNA turnover in SEM-Mb-treated cells. The negative control miRNA- and hsa-miR-99a-transfected cells were treated with 1.5 μM SEM-Mb for 24 h, and then incubated with 10 μg/mL actinomycin D for the indicated time periods. The level of NOX4 mRNA was analyzed by qRT-PCR (mean ± SD, * *p* < 0.05). NOX4 mRNA levels in cells not treated with actinomycin D were taken as 1.

As our data indicated the involvement of NOX4 in SEM-Mb-induced ROS production, we analyzed the effect of SEM-Mb treatment on NOX4 expression. The SEM-Mb increased NOX4 protein and mRNA expression without affecting the NOX4 promoter luciferase activity (Figure 2A–C). The SEM-Mb treatment increased the half-life of NOX4 mRNA as shown by the mRNA turnover assay (Figure 2D). The BAPTA-AM pretreatment abolished SEM-Mb-induced NOX4 upregulation (Figure 2E,F), whereas NAC had no inhibitory effect (Figure 2G). These results indicate that SEM-Mb induced Ca^2+^-mediated NOX4 expression. Accumulating evidence has shown that miRNAs induce translational repression and mRNA degradation [30]. Several miRNAs including miR-137, miR-25, miR-99a, and miR-146a are known to promote NOX4 mRNA decay [31,32,33]. Therefore, we analyzed the expression of these miRNAs. Among the four miRNAs, SEM-Mb reduced miR-99a expression (Figure 2H) and BAPTA-AM pretreatment abolished this suppression (Figure 2I). Transfection with hsa-miR-99a diminished the SEM-Mb-induced upregulation of NOX4 protein and mRNA levels (Figure 2J,K). Consistent with this, the miR-99a mimic inhibited the SEM-Mb-induced stabilization of NOX4 mRNA (Figure 2L). These results indicate that SEM-Mb induces Ca^2+^-mediated downregulation of miR-99a, leading to the upregulation of NOX4 expression.

### 2.4. The Cytotoxicity of SEM-Mb Is Related to ROS-Mediated p38 MAPK Activation

**Figure 3:**
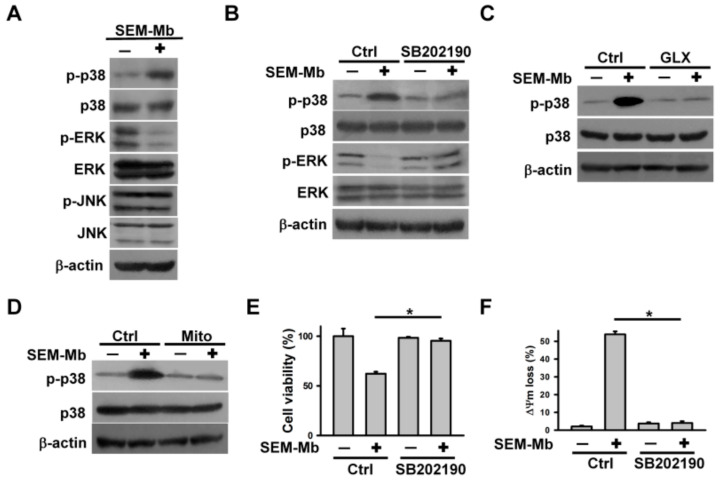
SEM-Mb triggered p38 MAPK phosphorylation by increasing mitochondrial ROS production. U937 cells were directly treated with 1.5 μM SEM-Mb for 24 h or pre-treated with 10 μM GLX351322, 10 μM Mito-TEMPO, or 10 μM SB202190 for 1 h and then incubated with 1.5 μM SEM-Mb for 24 h. (**A**) Western blot analyses of phosphorylated MAPKs in SEM-Mb-treated cells. (**B**) Effect of SB202190 on p-p38 MAPK and p-ERK levels in SEM-Mb-treated cells. Effect of (**C**) GLX351322 and (**D**) Mito-TEMPO on p-p38 MAPK levels in SEM-Mb-treated cells. (**E**) Effect of SB202190 on the viability of SEM-Mb-treated cells (mean ± SD, * *p* < 0.05). (**F**) Effect of SB202190 on SEM-Mb-induced ∆Ψm loss (mean ± SD, * *p* < 0.05).

Studies have indicated that MAPKs regulate apoptosis [34,35], and that Ca^2+^ and ROS signaling modulate MAPK phosphorylation [36,37]. Therefore, we analyzed the role of phosphorylated MAPK in SEM-Mb cytotoxicity. The SEM-Mb treatment increased p-p38 MAPK levels and reduced p-ERK levels; however, the levels of p-JNK remained unchanged (Figure 3A). Inhibition of p38 MAPK using SB202190 reversed the phosphorylation of p38 MAPK and ERK in SEM-Mb-treated cells (Figure 3B). In agreement, studies by Liu et al. [38] have reported inverse changes in p-p38 MAPK and p-ERK levels in U937 cells due to crosstalk between p38 MAPK and ERK. Pretreatment with NOX4 inhibitor or Mito-TEMPO abolished SEM-Mb-induced p38 MAPK activation (Figure 3C,D). Moreover, SB202190 inhibited SEM-Mb-induced cell death and ∆Ψm loss (Figure 3E,F). These findings highlight the causal relationship between ROS-mediated p38 MAPK activation and SEM-Mb-mediated cytotoxicity. 

### 2.5. SEM-Mb Promotes TNF-α Expression via A p38 MAPK-Dependent Post-Transcriptional Pathway

**Figure 4:**
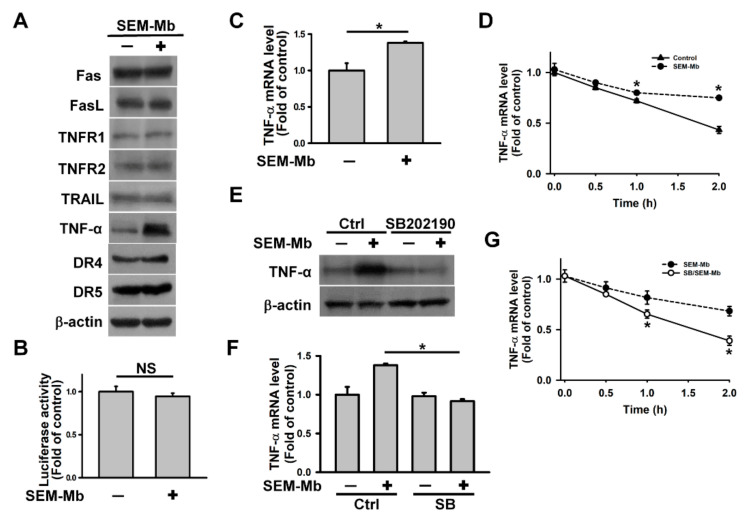
SEM-Mb-treated U937 cells showed increased TNF-α expression by reducing the decline in TNF-α mRNA. U937 cells were directly treated with 1.5 μM SEM-Mb for 24 h or pre-treated with 10 μM SB202190 for 1 h and then incubated with 1.5 μM SEM-Mb for 24 h. (**A**) Effect of SEM-Mb on TNF-α, TNFR1, TNFR2, TRAIL, DR4, DR5, Fas, and FasL protein expression. (**B**) Effect of SEM-Mb on the luciferase activity of the TNF-α promoter construct. TNF-α promoter construct-transfected cells were treated with 1.5 μM SEM-Mb for 24 h and then harvested for measuring luciferase activity (mean ± SD, NS, not statistically significant). (**C**) qRT-PCR analyses of TNF-α mRNA levels in SEM-Mb-treated cells (mean ± SD, * *p* < 0.05). (**D**) Effect of SEM-Mb treatment on TNF-α mRNA stability. Cells were treated with or without 1.5 μM SEM-Mb for 24 h, and then incubated with 10 μg/mL actinomycin D for the indicated time periods. The levels of TNF-α mRNA were analyzed by qRT-PCR (mean ± SD, * *p* < 0.05). (**E**) Effect of SB202190 on SEM-Mb-induced TNF-α expression. (**F**) Effect of SB202190 on TNF-α mRNA expression in SEM-Mb-treated cells (mean ± SD, * *p* < 0.05). (**G**) Effect of SB202190 on SEM-Mb-induced stabilization of TNF-α mRNA. U937 cells were pre-treated with 10 μM SB202190 for 1 h, and then incubated with 1.5 μM SEM-Mb for 24 h. SEM-Mb- and SB202190/SEM-Mb-treated cells were incubated with 10 μg/mL actinomycin D for the indicated time periods (mean ± SD, * *p* < 0.05).

As our results suggest that SEM-Mb cytotoxicity is associated with a death receptor-mediated pathway, we sought to analyze the effect of SEM-Mb treatment on the expression of TNF-α family proteins. The SEM-Mb increased TNF-α protein levels, but did not alter the expression of FasL, Fas, TRAIL, DR4, DR5, TNFR1, or TNFR2 (Figure 4A). Treatment with SEM-Mb did not affect TNF-α promoter luciferase activity but increased TNF-α transcript levels and slowed TNF-α mRNA decay (Figure 4B–D). SB202190 abrogated the effects of SEM-Mb on TNF-α expression and mRNA stabilization (Figure 4E–G). Collectively, our data confirmed that SEM-Mb promotes TNF-α expression via a p38 MAPK-dependent post-transcriptional pathway.

### 2.6. SEM-Mb-Induced p38 MAPK Activation Increases miR-29a Expression, Leading to the Inhibition of TTP Expression

**Figure 5:**
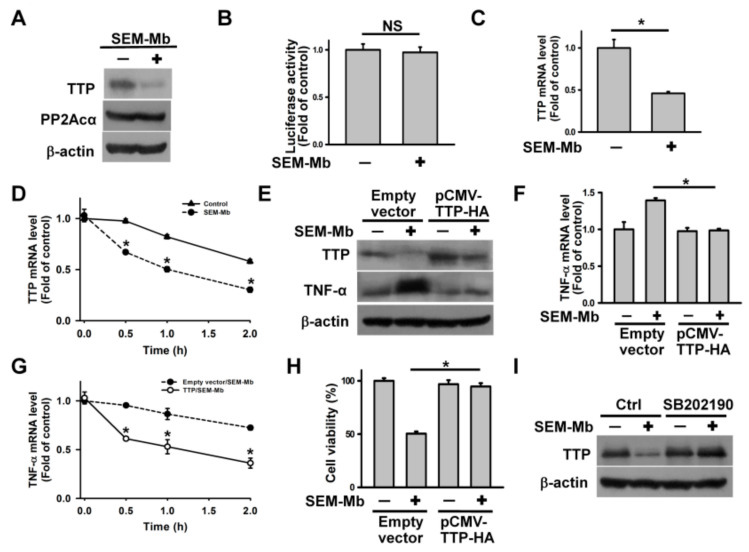

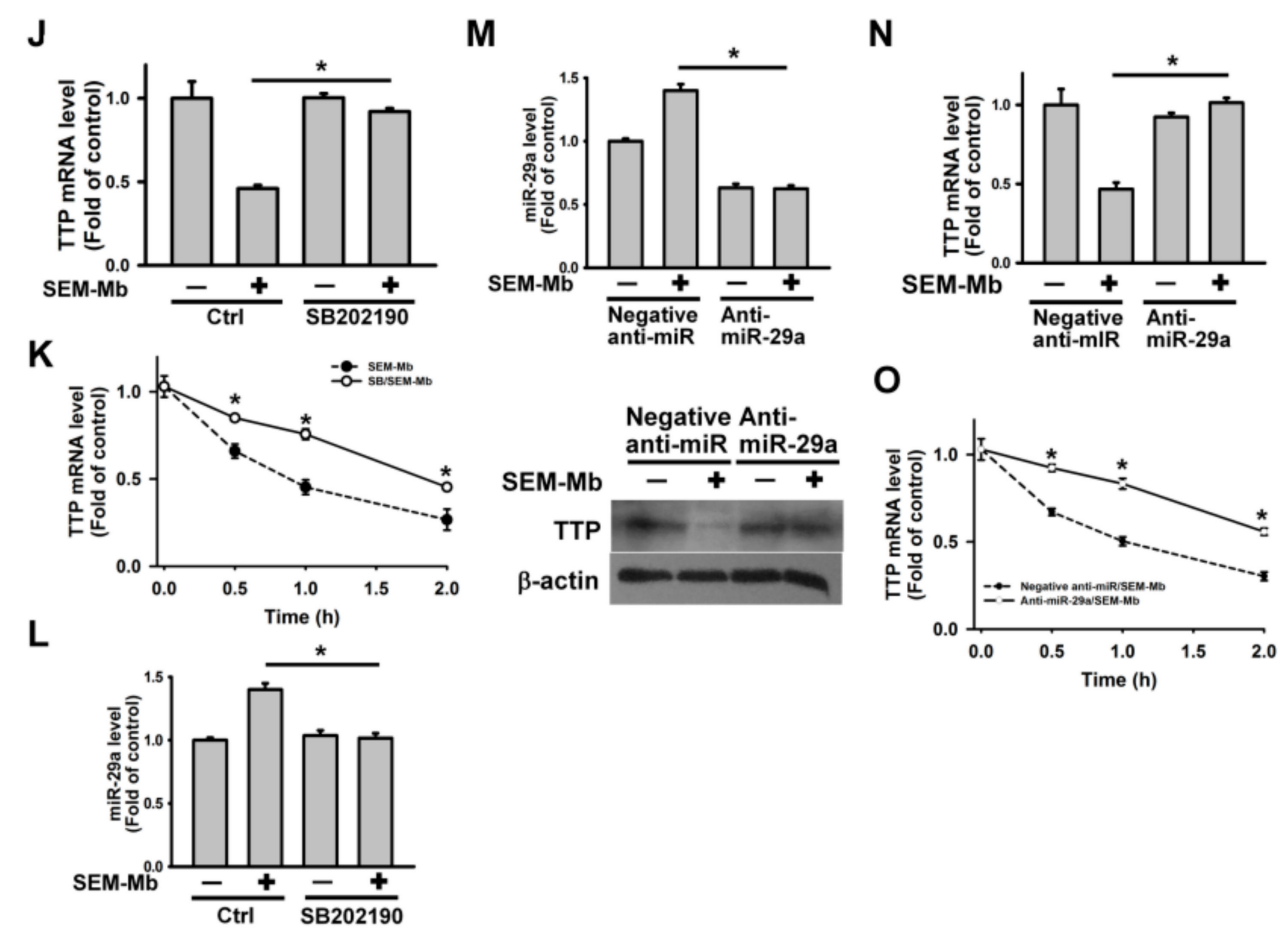
Inhibition of TTP expression by p38 MAPK prolonged the half-life of TNF-α mRNA in SEM-Mb-treated U937 cells. U937 cells were directly treated with 1.5 μM SEM-Mb for 24 h or pre-treated with 10 μM SB202190 for 1 h and then incubated with 1.5 μM SEM-Mb for 24 h. (**A**) Effect of SEM-Mb on PP2Acα and TTP protein expression. (**B**) Effect of SEM-Mb on the luciferase activity of TTP promoter. After transfection with pGL3-TTP for 24 h, the transfected cells were treated with 1.5 μM SEM-Mb for 24 h and then harvested for measuring luciferase activity (mean ± SD, NS, not statistically significant). (**C**) qRT-PCR analyses of TTP mRNA levels in SEM-Mb-treated cells (mean ± SD, * *p* < 0.05). (**D**) Effect of SEM-Mb treatment on TTP mRNA stability. Cells were treated with or without 1.5 μM SEM-Mb for 24 h, and then incubated with 10 μg/mL actinomycin D for the indicated time periods. The levels of TTP mRNA were analyzed by qRT-PCR (mean ± SD, * *p* < 0.05). (**E**) Overexpression of TTP attenuated SEM-Mb-induced TNF-α upregulation. After transfection with an empty vector or pCMV-TTP-HA for 24 h, the transfected cells were treated with 1.5 μM SEM-Mb for 24 h. (**F**) Effect of TTP overexpression on TNF-α mRNA levels in SEM-Mb-treated cells (mean ± SD, * *p* < 0.05). (**G**) Effect of SEM-Mb treatment on TNF-α mRNA stability in pCMV-TTP-HA-transfected cells. The empty vector- and pCMV-TTP-HA-transfected cells were treated with 1.5 μM SEM-Mb for 24 h, and then incubated with 10 μg/mL actinomycin D for the indicated time periods. The levels of TNF-α mRNA were analyzed by qRT-PCR (mean ± SD, * *p* < 0.05). (**H**) Overexpression of TTP rescued the viability of SEM-Mb-treated cells (mean ± SD, * *p* < 0.05). (**I**) Effect of SB202190 on TTP expression in SEM-Mb-treated cells. (**J**) Effect of SB202190 on TTP mRNA levels in SEM-Mb-treated cells. The levels of TTP mRNA were analyzed by qRT-PCR (mean ± SD, * *p* < 0.05). (**K**) Effect of SB202190 on TTP mRNA turnover in SEM-Mb-treated cells. U937 cells were pre-treated with 10 μM SB202190 for 1 h, and then incubated with 1.5 μM SEM-Mb for 24 h. SEM-Mb- and SB202190/SEM-Mb-treated cells were incubated with 10 μg/mL actinomycin D for the indicated time periods (mean ± SD, * *p* < 0.05). (**L**) Effect of SB202190 on the expression of miR-29a in SEM-Mb-treated cells (mean ± SD, * *p* < 0.05). (**M**) Transfection of anti-miR-29a inhibited SEM-Mb-induced TTP downregulation. Cells were transfected with negative control anti-miR or anti-miR-29a. After 24 h post-transfection, the cells were treated with 1.5 μM SEM-Mb for 24 h. (Top panel) qRT-PCR analysis of miR-29a levels in anti-miR-29a-transfected cells (mean ± SD, * *p* < 0.05). (Bottom panel) Western blot analysis of TTP expression in anti-miR-29a-transfected cells after SEM-Mb treatment. (**N**) Transfection of anti-miR-29a increased the expression of TTP mRNA in SEM-Mb-treated cells. The negative control anti-miR- and anti-miR-29a-transfected cells were treated with 1.5 μM SEM-Mb for 24 h. The levels of TTP mRNA were analyzed by qRT-PCR (mean ± SD, * *p* < 0.05). (**O**) Transfection of anti-miR-29a reduced TTP mRNA turnover in SEM-Mb-treated cells. The negative control anti-miR- and anti-miR-29a-transfected cells were treated with 1.5 μM SEM-Mb for 24 h, and then incubated with 10 μg/mL actinomycin D for the indicated time periods. The levels of TTP mRNA were analyzed by qRT-PCR (mean ± SD, * *p* < 0.05).

Liu et al. [39] discovered that hydroquinone-induced upregulation of protein phosphatase 2A catalytic subunit α (PP2Acα) promoted the degradation of tristetraprolin (TTP) in U937 cells, thereby slowing TTP-mediated TNF-α mRNA decay. We analyzed the expression of TTP and PP2Acα and observed that SEM-Mb treatment reduced TTP but not PP2Acα protein expression (Figure 5A). Additionally, SEM-Mb did not affect TTP promoter luciferase activity, but reduced TTP transcript levels and mRNA stability (Figure 5B–D). These findings suggest that SEM-Mb downregulates TTP expression through a post-transcriptional mechanism, rather than through proteasomal degradation. The SEM-Mb failed to induce TNF-α upregulation in cells ectopically expressing TTP (Figure 5E,F). Overexpression of TTP deprived SEM-Mb of the ability to stabilize TNF-α mRNA (Figure 5G) and induce cell death (Figure 5H). The SB202190 abrogated the ability of SEM-Mb to downregulate TTP expression and destabilize TTP mRNA (Figure 5I–K). A previous study revealed that miR-29a targets the 3′-UTR of TTP mRNA, thus suppressing TTP expression [40]. We examined whether miR-29a was involved in SEM-Mb-induced TTP suppression. The SEM-Mb treatment increased miR-29a expression, whereas SB202190 pretreatment inhibited SEM-Mb-induced events (Figure 5L). Transfection with anti-miR-29a impaired the ability of SEM-Mb to reduce TTP protein and mRNA expression (Figure 5M,N). Compared to the negative anti-miR control-transfected cells, anti-miR-29a-transfeced cells decreased the decline of TTP mRNA after SEM-Mb treatment (Figure 5O). These findings confirm that SEM-Mb-induced p38 MAPK activation increases miR-29a expression, leading to the inhibition of TTP expression.

### 2.7. SEM-Mb Triggers p38 MAPK/TTP Axis-Dependent TNF-α Expression by Promoting SIRT3 Degradation

**Figure 6:**
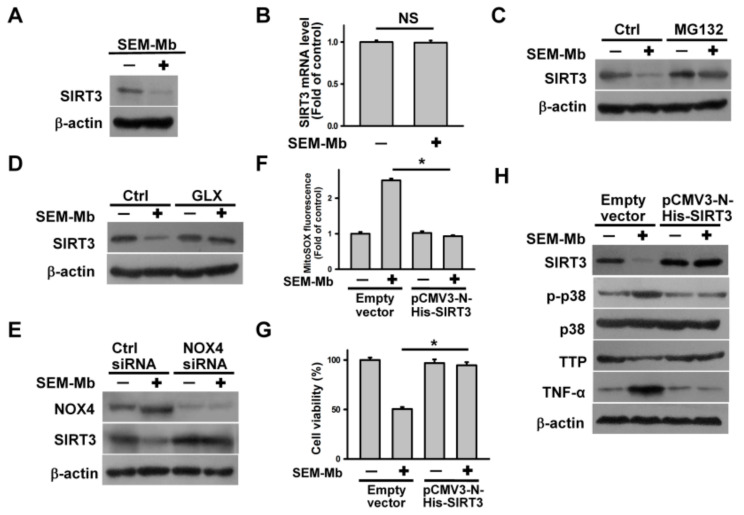
NOX4-mediated SIRT3 degradation regulated p38 MAPK/TTP axis-dependent TNF-α upregulation in SEM-Mb-treated cells. U937 cells were directly treated with 1.5 μM SEM-Mb for 24 h or pre-treated with 1 μM MG132 or 10 μM GLX351322 for 1 h and then incubated with 1.5 μM SEM-Mb for 24 h. (**A**) Effect of SEM-Mb on SIRT3 expression. (**B**) qRT-PCR analyses of SIRT3 mRNA levels in SEM-Mb-treated cells (mean ± SD, NS, not statistically significant). Effect of (**C**) MG132 and (**D**) GLX351322 on the SIRT3 expression in SEM-Mb-treated cells. (**E**) Effect of NOX4 depletion on SIRT3 expression in U937 cells. U937 cells were transfected with 100 nM negative control siRNA or NOX4 siRNA, respectively. After 24 h post-transfection, the cells were treated with 1.5 μM SEM-Mb for 24 h. (**F**) Effect of SIRT3 overexpression on SEM-Mb-induced the production of mitochondrial ROS in U937 cells (mean ± SD, * *p* < 0.05). U937 cells were transfected with empty vector or pCMV3-N-His-SIRT3, respectively. After 24 h post-transfection, the transfected cells were treated with 1.5 μM SEM-Mb for 24 h. (**G**) Effect of SIRT3 overexpression on the viability of SEM-Mb-treated cells (mean ± SD, * *p* < 0.05). (**H**) Effect of SIRT3 overexpression on the expression of p-p38 MAPK, TTP, and TNF-α in SEM-Mb-treated U937 cells.

Previous studies have indicated that SIRT3 inhibition promotes mitochondrial ROS production in U937 cells [41]. Therefore, we analyzed the effect of SEM-Mb treatment on SIRT3 expression. The SEM-Mb treatment reduced SIRT3 protein levels but not mRNA expression (Figure 6A,B). Pretreatment with MG132 abrogated SEM-Mb-induced SIRT3 downregulation (Figure 6C), indicating that SEM-Mb treatment promotes SIRT3 degradation. Inhibition or depletion of NOX4 diminished the SEM-Mb-induced downregulation of SIRT3 (Figure 6D,E), indicating that NOX4 suppresses SIRT3 expression. The enforced expression of SIRT3 alleviated SEM-Mb-induced mitochondrial ROS production and cell death (Figure 6F,G). Compared to cells transfected with the empty vector, SEM-Mb failed to alter the expression of p-p38 MAPK, TNF-α, and TTP in pCMV-N-His-SIRT3-transfected cells (Figure 6H). These findings confirmed that SEM-Mb elicited mitochondrial ROS generation by promoting SIRT3 degradation, thereby triggering p38 MAPK/TTP axis-dependent TNF-α expression.

### 2.8. SEM-Mb Induces Apoptosis in HL-60 Cells via NOX4/SIRT3/p38 MAPK/TTP Axis-Dependent TNF-α Expression

**Figure 7:**
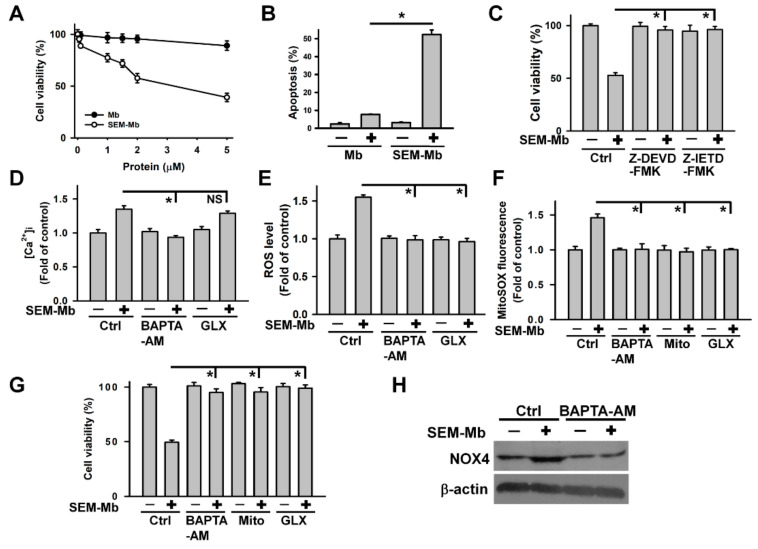

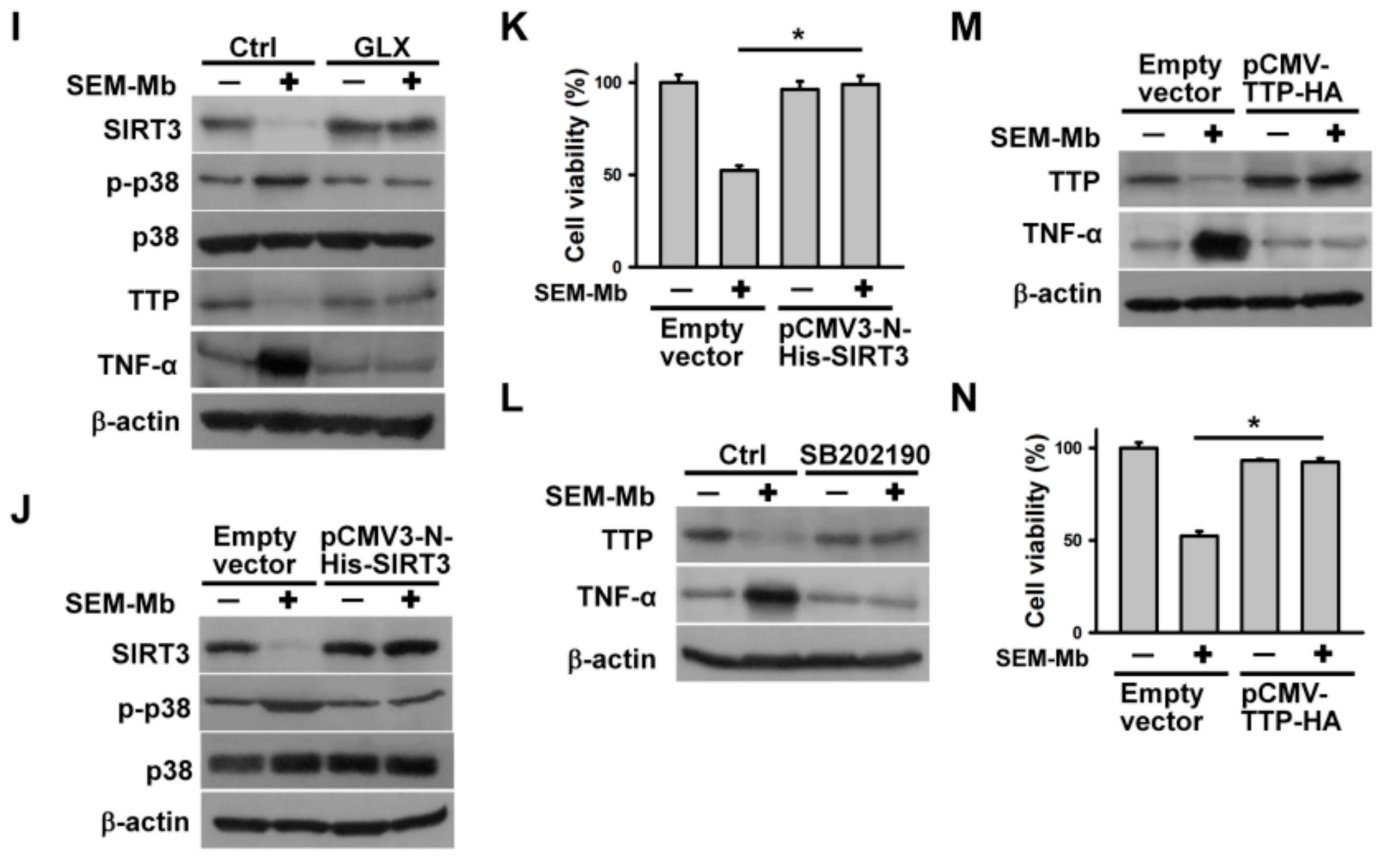
SEM-Mb-induced HL-60 cell death was associated with increased TNF-α expression triggered by the NOX4/SIRT3/p38 MAPK/TTP axis. Without specific indication, HL-60 cells were treated with 2 μM SEM-Mb for 24 h. HL-60 cells were pre-treated with 10 μM Z-DEVD-FMK, 10 μM Z-IETD-FMK, 10 μM BAPTA-AM, 10 μM GLX351322, 10 μM Mito-TEMPO, or 10 μM SB202190 for 1 h, and then incubated with 2 μM SEM-Mb for 6 h (for measuring ROS levels and MitoSOX fluorescence) or 24 h (for measuring [Ca^2+^]i and cell viability). (**A**) SEM-Mb induced cell death in a concentration-dependent manner. HL-60 cells were incubated with varying concentrations of SEM-Mb or Mb for 24 h. Cell viability was determined by MTT assay. Results are expressed as the percentage of cell survival relative to the control. Each value is the mean ± SD of triplicate determinations. (**B**) Flow cytometry analysis of SEM-Mb-induced apoptosis in HL-60 cells. Apoptosis was assessed in triplicate by annexin V/propidium iodide double staining followed by flow cytometry, and percentage apoptosis is shown as percentage of annexin V-positive cells (mean ± SD, * *p* < 0.05). (**C**) Effect of caspase inhibitors on SEM-Mb-induced cell death (mean ± SD, * *p* < 0.05). (**D**) Effect of BAPTA-AM and GLX351322 on the [Ca^2+^]i level in SEM-Mb-treated cells (mean ± SD, * *p* < 0.05, NS, not statistically significant). (**E**) Effect of BAPTA-AM and GLX351322 on SEM-Mb-induced ROS generation (mean ± SD, * *p* < 0.05). (**F**) Effect of BAPTA-AM, Mito-TEMPO, and GLX351322 on mitochondrial ROS levels in SEM-Mb-treated cells (mean ± SD, * *p* < 0.05). (**G**) Effect of BAPTA-AM, Mito-TEMPO, and GLX351322 on the viability of SEM-Mb-treated cells (mean ± SD, * *p* < 0.05). (**H**) Effect of BAPTA-AM on SEM-Mb-induced NOX4 expression. (**I**) Effect of GLX351322 on the expression of SIRT3, p-p38 MAPK, TTP, and TNF-α in SEM-Mb-treated cells. (**J**) Transfection of pCMV3-N-His-SIRT3 inhibited SEM-Mb-induced p38 MAPK phosphorylation. HL-60 cells were transfected with empty vector or pCMV3-N-His-SIRT3, respectively. After 24 h post-transfection, the transfected cells were treated with 2 μM SEM-Mb for 24 h. (**K**) Effect of SIRT3 overexpression on the viability of SEM-Mb-treated cells (mean ± SD, * *p* < 0.05). (**L**) Effect of SB202190 on the expression of TTP and TNF-α in SEM-Mb-treated cells. (**M**) Effect of TTP overexpression on SEM-Mb-induced TNF-α expression. After transfection with an empty vector or pCMV-TTP-HA for 24 h, the transfected cells were treated with 2 μM SEM-Mb for 24 h. (**N**) Effect of TTP overexpression on the viability in SEM-Mb-treated HL-60 cells (mean ± SD, * *p* < 0.05).

To further examine whether SEM-Mb exhibited cytotoxicity in other leukemia cells through a similar pathway, we investigated its cytotoxicity in other leukemia cells. Daunorubicin and cytarabine are standard drugs for the treatment of acute myeloid leukemia [42,43]. Some studies have shown that, compared to U937 cells, HL-60 cells are more resistant to daunorubicin and cytarabine, owing to which HL-60 cells highly expressed myeloperoxidase [44,45]. Therefore, we investigated the cytotoxicity of SEM-Mb in leukemia HL-60 cells. Unlike Mb, SEM-Mb was cytotoxic to HL-60 cells with an IC50 value of approximately 2 μM (Figure 7A). The SEM-Mb treatment induced apoptosis in HL-60 cells (Figure 7B), whereas caspase inhibitors maintained cell viability, regardless of SEM-Mb treatment (Figure 7C). Use of BAPTA-AM, but not GLX351322, inhibited the SEM-Mb-induced [Ca^2+^]i elevation (Figure 7D). Both BAPTA-AM and GLX351322 abrogated cellular and mitochondrial ROS production in SEM-Mb-treated cells and protected HL-60 cells from SEM-Mb cytotoxicity (Figure 7E–G). Pretreatment with Mito-TEMPO attenuated SEM-Mb-induced mitochondrial ROS production and cell death (Figure 7F,G). The SEM-Mb-triggered NOX4 upregulation was alleviated by pretreatment with BAPTA-AM (Figure 7H). Collectively, our data indicate that SEM-Mb stimulated mitochondrial ROS production in HL-60 cells through Ca^2+^-mediated NOX4 expression. The SEM-Mb decreased SIRT3 and TTP expression but increased p38 MAPK and TNF-α levels, whereas GLX351322 inhibited SEM-Mb-induced effects (Figure 7I). Transfection with pCMV3-N-His-SIRT3 eliminated the SEM-Mb-induced p38 MAPK phosphorylation and cell death (Figure 7J,K). Pretreatment with SB202190 increased TTP expression and decreased TNF-α expression in HL-60 cells treated with SEM-Mb (Figure 7L). Overexpression of TTP attenuated the induction of TNF-α expression and cell death induced by SEM-Mb (Figure 7M,N). These results confirm that SEM-Mb induces apoptosis in HL-60 cells via NOX4/SIRT3/p38 MAPK/TTP axis-dependent TNF-α expression.

### 2.9. SEM-Mb Induces Death of THP-1, K562, Jurkat, and ABT-199-Resistant U937 Cells via p38 MAPK-Mediated TNF-α Expression

In addition to U937 and HL-60 cells, the cytotoxicity of SEM-Mb on AML THP-1, chronic myeloid leukemia (CML) K562, and acute lymphoblastic leukemia (ALL) Jurkat cells was analyzed. The IC50 values of SEM-Mb on THP-1, K562, and Jurkat cells were 2 μM, 1.5 μM, and 1.5 μM, respectively (Supplementary Figure S2A). Treatment with SEM-Mb induced apoptosis of these cells (Supplementary Figure S2B). Furthermore, SEM-Mb induced p38 MAPK activation and TNF-α upregulation in THP-1, K562, and Jurkat cells (Supplementary Figure S2C). Pretreatment with SB202190 inhibited SEM-Mb induced apoptosis and TNF-α upregulation, indicating that SEM-Mb-induced death of THP-1, K562, and Jurkat cells occurs through p38 MAPK-dependent TNF-α expression. The BCL2 inhibitor ABT-199 has been used to treat chronic lymphocytic leukemia and AML [46]. However, ABT-199-induced upregulation of MCL1 expression confers resistance to this drug [46]. We further examined whether SEM-Mb potentially induced the death of ABT-199-resistant U937 cells and observed that the cytotoxicity of SEM-Mb to ABT-199-resistant U937 cells was related to p38 MAPK-dependent TNF-α expression (Supplementary Figure S2A–C). Collectively, these findings indicate that SEM-Mb is cytotoxic to other leukemia cells.

## 3. Discussion

**Figure 8:**
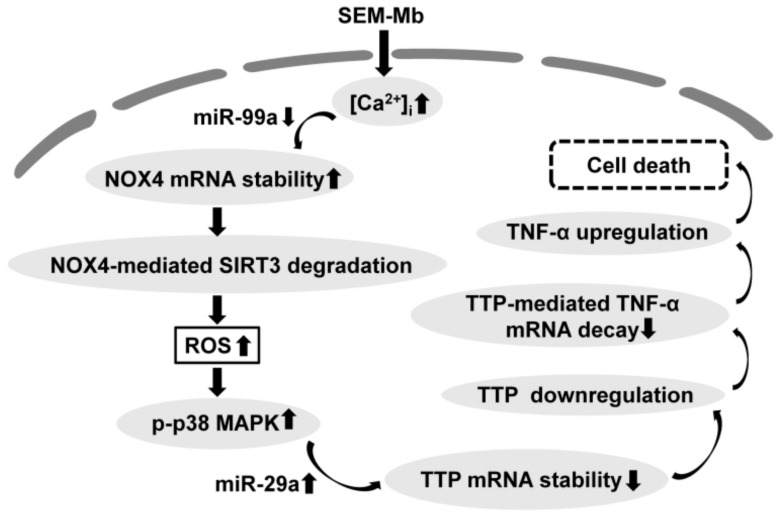
SEM-Mb induced TNF-α-mediated cell death signaling in U937 and HL-60 cells. SEM-Mb induces Ca^2+^-mediated miR-99a downregulation, leading to upregulate NOX4 expression in U937 cells. NOX4-induced SIRT3 degradation promotes mitochondrial ROS generation, which in turn elicits p38 MAPK-mediated miR-29a expression, thereby inhibiting TTP expression. Because TTP destabilizes TNF-α mRNA, SEM-Mb-induced TTP downregulation increases TNF-α expression. SEM-Mb treatment also induces TNF-α upregulation in HL-60 cells through the Ca^2+^/NOX4/SIRT3/p38 MAPK/TTP axis. Consequently, SEM-Mb-induced TNF-α upregulation triggers apoptosis in U937 and HL-60 cells.

Our data indicate that SEM-Mb induces Ca^2+^-mediated miR-99a downregulation, resulting in increased NOX4 expression in U937 cells. The NOX4-triggered SIRT3 degradation promotes mitochondrial ROS/p38 MAPK-mediated miR-29a expression, thereby downregulating TTP expression. As TTP destabilizes TNF-α mRNA, SEM-Mb-induced TTP downregulation increases TNF-α expression (Figure 8). Consequently, SEM-Mb activates the TNF-α-mediated death pathway in U937 cells. Abolishment of TNF-α upregulation protected U937 cells from SEM-Mb-induced cell death, indicating a causal role of TNF-α in SEM-Mb-mediated cytotoxicity. A similar mechanism also explains SEM-Mb-induced death of HL-60 cells. In addition, p38 MAPK-mediated TNF-α expression was associated with cytotoxicity of SEM-Mb against THP-1, K562, Jurkat, and ABT-199-resistant U937 cells. Our data showed that SEM-Mb effectively inhibited the viability of chemo-resistant leukemia cells. Recent studies have revealed that docetaxel induces SIDT2-mediated lysosomal degradation of miR-25, resulting in decreased NOX4 mRNA decay [47]. However, the depletion of SIDT2 using siRNA did not increase miR-99a expression in SEM-Mb-treated U937 cells (data not shown). Therefore, the mechanism underlying SEM-Mb-induced miR-99a downregulation remains unclear. Compelling evidence indicates that SIRT3-mediated deacetylation is essential for the activity of SOD2 to remove mitochondrial ROS [48,49]. Shanmugasundaram et al. [50] demonstrated that NOX4 modulates mitochondrial ROS production. Consistent with these findings, our data showed that SEM-Mb-induced NOX4 expression promotes SIRT3 degradation, thereby increasing mitochondrial ROS production. A previous study indicated that Skp2, an E3 ubiquitin protein ligase, modulates SIRT3 degradation [51]. Pretreatment with SZL P1-41 (an Skp2 inhibitor) did not affect the SEM-Mb-induced SIRT3 downregulation (data not shown). Therefore, the mechanism underlying NOX4-elicited SIRT3 degradation remains elusive. Wang et al. [41] demonstrated that albendazole induces TTP degradation in U937 cells by triggering p38 MAPK-dependent TTP phosphorylation. Other studies have indicated that hydroquinone induces p38 MAPK/PP2A axis-dependent TTP degradation in U937 cells [47]. The data in this study revealed that p38 MAPK-mediated miR-29a expression destabilizes TTP mRNA in U937 cells after SEM-Mb treatment. Taken together, these results suggest that the regulatory mechanism of p38 MAPK in suppressing TTP expression is stimulus-dependent. Notably, previous studies have shown that, in addition to apoptosis, increased ROS production triggers necroptosis or ferroptosis [52]. Although necroptosis can be activated by the ligand binding to TNF family receptors, activated caspase-8 is not involved in the necroptosis pathway [53]. On the other hand, the mechanism of ferroptosis is independent of the caspase-mediated pathway [54]. As the cytotoxicity of SEM-Mb is related to the activation of caspase-8/-3 (Figure 1D), it is conceivable that SEM-Mb-induced cell death is not mediated through necroptosis or ferroptosis.

The heme oxygenase activity of Mb catalyzes the production of cellular ROS [55,56]. Because SEM-Mb lacks a heme group as shown by the loss of the Soret peak, we hypothesized that SEM-Mb-induced ROS generation is independent of heme oxygenase activity. Braganza et al. [57] argued that the heme group of Mb is essential for Mb-induced inhibition of breast cancer cell proliferation and that the growth inhibitory mechanism of intracellularly expressed Mb is achieved through the inhibition of mitochondrial fission. Our results showed that extracellular treatment with SEM-Mb, but not Mb, activated the TNF-α-mediated death pathway in leukemia cells. Zager and Burkhart [24] found that Mb treatment induced the death of the human proximal tubular cell line HK-2. Other studies have suggested that megalin- and cubilin-mediated endocytosis of Mb in the renal proximal tubule leads to nephrotoxicity [58]. Therefore, the possibility that internalization of SEM-Mb modulates its cytotoxicity warrants further investigation. Notably, treatment with 10 mg/mL (~588 μM) Mb for 24 h resulted in an approximately 65–85% reduction in the viability of HK2 cells [24]. Our results indicate that SEM-Mb at a concentration of ≤2.0 μM considerably reduced the viability of U937 and HL-60 cells. It is evident that the modification of the carboxyl groups renders Mb more cytotoxic. Some studies have indicated that free heme released from hemoproteins induces TNF-α-dependent death in macrophages [59]. Given that SEM-Mb does not contain a heme group, we believe that the mechanism by which SEM-Mb induces TNF-α upregulation in leukemia cells differs from that of heme in macrophages.

Compelling evidence indicates that oleic acid stabilizes the molten globular conformation of apo-LA, which is essential for the cytotoxicity of apo-LA-oleic acid complexes [14,60]. The structure of apo-Mb has a molten globule-like character [61], and lipid-protein complexes formed by apo-Mb and oleic acid also show antitumor activity [62]. Notably, carboxyl group-modified LA with a molten globule conformation exhibits cytotoxicity in U937 and breast cancer MCF-7 cells [11,12]. Together with the finding that modification of the carboxyl groups causes dramatic distortion of the Mb structure [25], our results underscore the idea that conformational changes are related to SEM-Mb cytotoxicity. Previous studies have indicated that the apo-Mb 56-131 peptide exhibits membrane-perturbing activity in physiological solutions, whereas apo-Mb and Mb failed to show this activity [17]. More importantly, the apo-Mb 56-131 peptide loses the characteristic α-helical structure of apo-Mb and Mb [17]. These results indicate that the inherent activity of the peptide fragment at positions 56-131 was hindered by the intact protein structure of Mb and apo-Mb. Although the data from this study revealed that SEM-Mb is cytotoxic to leukemia cells, the application of SEM-Mb as a tumoricidal agent requires further study to evaluate its feasibility. Importantly, the potential immunogenicity of SEM-Mb for inducing antibodies against SEM-Mb in vivo should be considered. Several studies have shown that the repeated use of protein pharmaceuticals induces antibodies against these agents, thereby reducing their efficacy [63,64]. To eliminate this possibility, a viable idea is to decipher the active region in SEM-Mb responsible for anti-leukemia activity and then use this active region to develop peptidomimetics. Notably, El-Maksound et al. [65] reported that milk protein-mucilage complexes formed by milk proteins with Isabgol husk mucilage or *Ziziplus spina-christi* mucilage enhanced the anti-cancer activity of milk proteins. As reducing the dose and frequency of using SEM-Mb can delay its potential antibody induction, complexation of SEM-Mb with natural components may be a feasible strategy to increase its anti-leukemia activity at a reduced dose.

## 4. Materials and Methods

### 4.1. Chemicals

Myoglobin (Mb) from equine skeletal muscle, semicarbazide, N-acetylcysteine (NAC), actinomycin D, SB202190, 3-(4,5-dimethylthiazol-2-yl)-2,5-diphenyl tetrazolium bromide (MTT), Mito-TEMPO, MG132, and HRP-linked secondary antibodies were purchased from Sigma-Aldrich Inc. (St. Louis, MO, USA), and Z-DEVD-FMK and Z-IETD-FMK were from Calbiochem (San Diego, CA, USA). The ML171 and GLX351322 were obtained from MedChem Express (Monmouth Junction, NJ, USA), and H_2_DCFDA, rhodamine 123, Fluo-4 AM, MitoSOX Red, and BAPTA-AM were from Thermo Fisher Scientific (Waltham, MA, USA).

### 4.2. Modification of Mb with Semicarbazide

Chemical modification of the carboxyl groups in Mb with semicarbazide (Supplementary Figure S3) was conducted according to the procedure described in [25]. A MALDI-TOF analysis showed that there were approximately 19 semicarbazide-conjugated carboxyl groups in the SEM-Mb. Measurement of circular dichroism spectra indicated that the carboxyl group modification profoundly distorted the secondary structure of Mb [25].

### 4.3. Cell Culture

Human AML U937, AML HL-60, AML THP-1, CML K562, and ALL Jurkat cells from BCRC (Hsinchu, Taiwan) were grown in RPMI-1640 medium supplemented with 10% FCS, 2 mM L-glutamine, 1% sodium pyruvate, and 1% streptomycin/penicillin. Cells were maintained in a humidified incubator with a 95% air/5% CO_2_ atmosphere at 37 °C. Cell viability was detected using a MTT assay [66]. Cell apoptosis was detected by staining cells with annexin V-FITC kit (Thermo Fisher Scientific) followed by flow cytometric analysis. The ABT-199-resistant U937 cells were prepared according to the procedure described in our previous studies [67]. Cell culture supplements and media were the products of GIBCO/Life Technologies Inc. (Carlsbad, CA, USA). The morphologies of U937, HL-60, THP-1, K562, Jurkat, and ABT-199-resistant U937 cells are shown in Supplementary Figure S4.

### 4.4. Detection of ROS, [Ca^2+^]i, and Mitochondrial Depolarization

Intracellular and mitochondria ROS levels were detected using H_2_DCFDA and MitoSOX Red, respectively. Rhodamine 123 and Fluo-4 AM were employed to measure mitochondrial membrane potential (∆Ψm) and [Ca^2+^]i, respectively. After treatment in the indicated conditions, cells were incubated with 10 μM H_2_DCFDA, 5 μM MitoSOX, 1 mM Fluo-4 AM, or 20 nM rhodamine 123 for 15 min. Afterwards, cells were washed in phosphate-buffered saline, and the fluorescent signal was measured using flow cytometry. 

### 4.5. Immunoblotting

Immunoblotting was performed essentially as previously described [68]. The primary antibodies used for immunoblotting were shown as following: β-actin (Sigma-Aldrich Inc., St. Louis, MO, USA), BCL2L1, BID (BD Pharmingen Technical, San Jose, CA, USA), BAX, BAK, BCL2, SIRT3, TNFR2, DR4, DR5, JNK, p38 MAPK, ERK, p-JNK, p-p38 MAPK, p-ERK, caspase-3, caspase-8 (Cell Signaling Technology, Beverly, MA, USA), NOX4 (Novus Biologicals, Centennial, CO, USA), FADD, TNF-α, FasL, Fas, PP2Acα, MCL1, tristetraprolin (TTP), PARP, TRAIL (Santa Cruz Biotechnology, Santa Cruz, CA, USA), and TNFR1 (R&D Systems, Minneapolis, MI, USA). Protein bands visualization was done with enhanced chemiluminescence substrate (Perkin Elmer. Waltham, MA, USA). Each immunoblot was performed no less than three times.

### 4.6. qRT-PCR

The RNeasy mini kit (QIAGEN, Leiden, The Netherlands) was used to extract total cellular RNA. The M-MLV reverse transcriptase (Promega, Madison, WI, USA) was used to synthesize cDNAs. The qPCR was conducted using GoTaq qPCR Master mix (Promega). Gene expression was calculated using 2^−ΔΔCt^ method relative to GAPDH mRNA levels. The PCR primer sequences are shown in Supplementary Table S1. To determine TNF-α, TTP, and NOX4 mRNA half-lives, transcription was inhibited by incubation with 10 μg/mL actinomycin D for the indicated time periods.

### 4.7. Transfection with Plasmid and siRNA

The plasmids pCMV3-N-His-SIRT3 and pCMV-TTP-HA were described in our previous studies [41]. Gene silencing experiments were performed using FADD siRNA, NOX4 siRNA, and negative control siRNA (Santa Cruz Biotechnology). TurboFect™ Transfection Reagent (Thermo Fisher Scientific) was used to transfect plasmids and siRNAs into cells.

### 4.8. Stem-Loop qRT-PCR for miRNAs

Reverse transcription of miRNAs was performed using ImProm-II Reverse Transcription System (Promega) and stem-loop reverse transcription (RT) primer. The expression levels of miRNAs were analyzed using GoTaq qPCR Master mix (Promega). Stem-loop RT primers and qPCR primers for miRNAs are listed in Supplementary Table S2.

### 4.9. Transient Transfection of miRNA Mimic and Inhibitor

Ambion^TM^ anti-miR-29a, hsa-miR-99a, negative control miR, and negative anti-miR control from Life Technologies Corporation (Carlsbad, CA, USA) were transfected into cells using TurboFect™ Transfection Reagent (Thermo Fisher Scientific). After 24 h post-transfection, the cells were treated with SEM-Mb for 24 h.

### 4.10. Luciferase Assay

The luciferase construct pGL3-TNF-α and pGL3-NOX4 were described elsewhere [12,69]. The human TTP promoter region containing nucleotides −1345 to +66 was subcloned into pGL3-basic vector (Promega) between *Sac*I and *Xho*I sites. TurboFect™ Transfection Reagent (Thermo Fisher Scientific) was used to transfect the promoter constructs into cells. Luciferase activity was measured using dual-luciferase reporter assay system kit (Promega). To normalize the transfection efficiency, the luciferase signal was calculated using the *Renilla* luciferase signal as an internal control.

### 4.11. Statistical Analysis

Results are expressed as the mean ± SD of at least three independent experiments. The statistical significance of differences between two groups was evaluated using Student’s *t*-test. Comparison of multiple groups was performed using one-way ANOVA with Tukey’s test. *p* value of < 0.05 was defined statistically significant.

## 5. Conclusions

Our data reveal that chemical modification of the carboxyl groups with semicarbazide enables Mb to show cytotoxicity and demonstrate that the chemically modified Mb activates TNF-α-mediated apoptosis in U937 cells through a NOX4/SIRT3/p38 MAPK/TTP axis-mediated pathway. A similar pathway also describes the cytotoxicity of semicarbazide-modified Mb in HL-60, THP-1, K562, Jurkat, and ABT-199-resistant U937 cells. Notably, Mb does not show cytotoxic activity prior to modification of the carboxyl group. Together with the finding that carboxyl group modification leads to conformational distortion in Mb [25], the data from this study highlight that chemical modification is a viable strategy for exploring intrinsic activities hindered by the conformation of protein. Furthermore, our data suggest that the carboxyl group-modified Mb is a potential structural template for the generation of tumoricidal proteins.

## Data Availability

Data is contained within the article and supplementary materials.

## Supplementary Materials

The following supporting information can be downloaded at https://www.mdpi.com/article/10.3390/ph15091066/s1. Figure S1. Treatment of U937 cells with SEM-Mb increased intracellular Ca2+ concentration and ROS levels; Figure S2. SEM-Mb induced death of THP-1, K562, Jurkat, and ABT-199-resistant U937 cells via p38 MAPK-mediated TNF-α expression; Figure S3. Schematic showing the modification of the carboxyl group in Mb with semicarbazide; Figure S4. The morphologies of (A) U937, (B) ABT-199-resistant U937 cells, (C) HL-60, (D) THP-1, (E) Jurkat, and (F) K562 cells; Table S1. Primers used for qRT-PCR; Table S2. Primers used for qRT-PCR of miRs.
