# Supplementary material for: Carboxyl Group-Modified Myoglobin Induces TNF-α-Mediated Apoptosis in Leukemia Cells
Source: Pharmaceuticals (Basel). 2022 Aug 27;15(9):1066. doi: 10.3390/ph15091066 (PMC9501283; doi:10.3390/ph15091066)
Supplement: Supplementary file 1 [file pharmaceuticals-15-01066-s001.zip › pharmaceuticals-1887690-supplementary.pdf]

Table S1. Primers used for qRT-PCR.

Gene	Nucleotide sequence
NOX4 (forward)	5′-GGCTGGAGGCATTGGAGTAA-3′
NOX4 (reverse)	5′-CCAGTCATCCAACAGGGTGTT-3′
SIRT3 (forward)	5′-GACATTCGGGCTGACGTGATGGC-3′
SIRT3 (reverse)	5′-CAACCACATGCAGCAAGAACCTCTG-3′
TTP (forward)	5′-CATCCACAACCCTAGCGAAGACCTG-3′
TTP (reverse)	5′-CAGAGAAGGCAGAGGGTGACAGTG-3′
TNF-α (forward)	5′-CTGGAGAAGGGTGACCGACTCAG-3′
TNF-α (reverse)	5′-TAGACCTGCCCAGACTCGGCAAAG-3′
GAPDH (forward)	5'-GAAATCCCATCACCATCTTCCAGG-3'
GAPDH (reverse)	5'-GAGCCCCAGCCTTCTCCATG-3'

Table S2. Primers used for qRT-PCR of miRs.

miRs	Nucleotide sequence
miR-25	
Stem loop	5'-GTCGTATCCAGTGCAGGGTCCGAGGTATTCGCAC TGGATACGACTCAGAC-3'
Forward	5'-GTATACCATTGCACTTGTCTC-3'
Reverse	5'-GTGCAGGGTCCGAGGT-3'
miR-146a	
Stem loop	5'-GTCGTATCCAGTGCAGGGTCCGAGGTATTCGCAC TGGATACGACAACCCA-3'
Forward	5'-GTATACTGAGAACTGAATTCC-3'
Reverse	5'-GTGCAGGGTCCGAGGT-3'
miR-99a	
Stem loop	5'-GTCGTATCCAGTGCAGGGTCCGAGGTATTCGCAC TGGATACGACCACAAG-3'
Forward	5'-GTATACAACCCGTAGATCCGA-3'
Reverse	5'-GTGCAGGGTCCGAGGT-3'
miR-137	
Stem loop	5'-GTCGTATCCAGTGCAGGGTCCGAGGTATTCGCAC TGGATACGACCTACGC-3'
Forward	5'-GTATACTTATTGCTTAAGAAT-3'
Reverse	5'-GTGCAGGGTCCGAGGT-3'
miR-29a	
Stem loop	5'-GTTGGCTCTGGTGCAGGGTCCGAGGTATTCGCAC CAGAGCCAACTAACCG -3'
Forward	5'-GTTTGGTAGCACCATCTGAAAT-3'
Reverse	5'-GTGCAGGGTCCGAGGT-3'

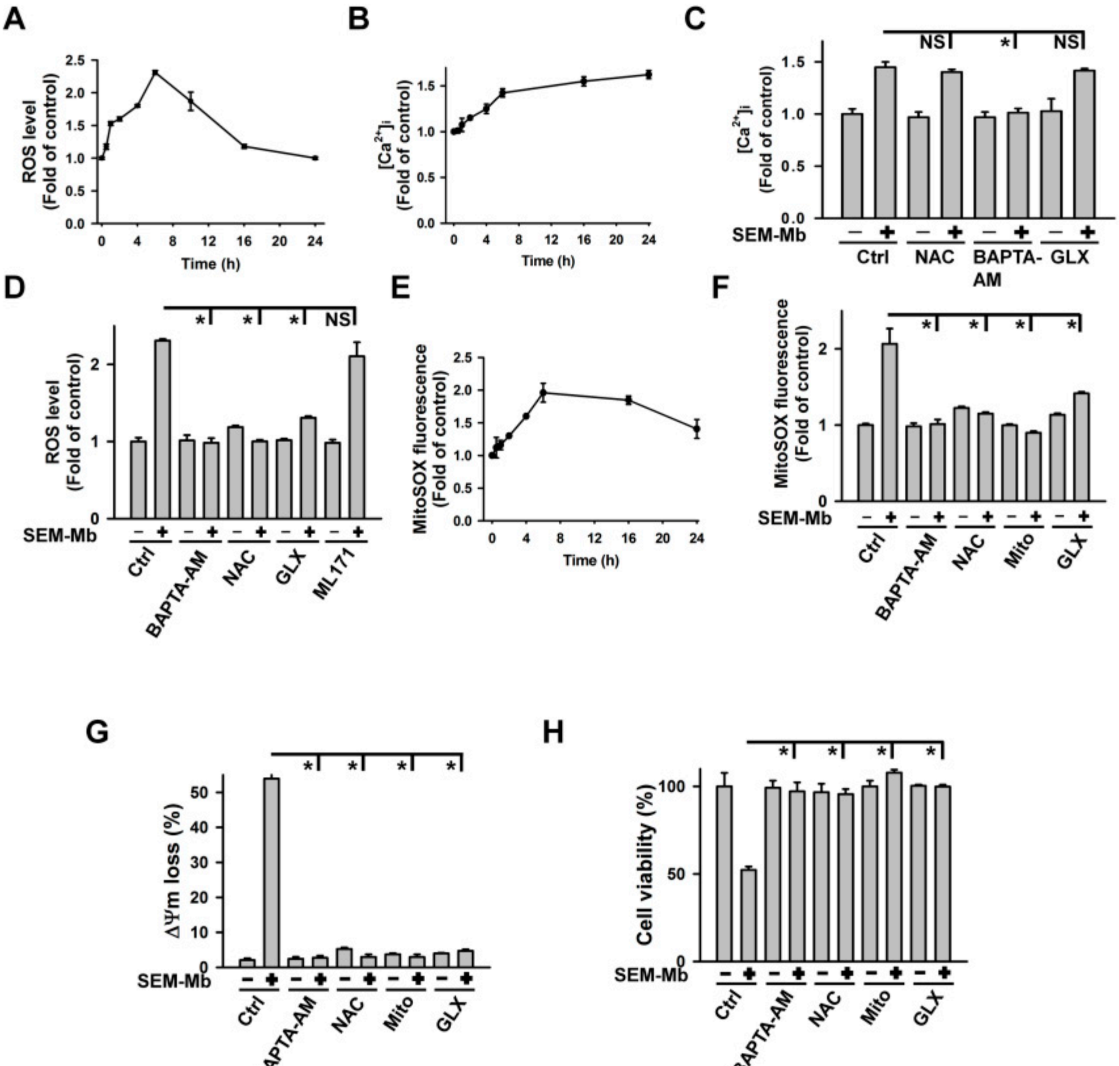

Figure S1. Treatment of U937 cells with SEM-Mb increased intracellular Ca^{2+} concentration and ROS levels. U937 cells were pre-treated with 2 mM N-acetylcysteine (NAC), 10 μM BAPTA-AM, 10 μM GLX351322, 10 μM ML171, or 10 μM Mito-TEMPO for 1 h, and then incubated with 1.5 μM SEM-Mb for 6 h (for measuring ROS level and MitoSOX fluorescence) or 24 h (for measuring $[Ca^{2+}]i$, cell viability, and ΔΨm). (A) SEM-Mb induced ROS generation in U937 cells. U937 cells were treated with 1.5 μM SEM-Mb for indicated time periods. Results were shown as fold-increase in fluorescence intensity compared with the control group. Each value is the mean±SD of three independent experiments with triplicate measurements. (B) SEM-Mb induced an elevation of $[Ca^{2+}]i$ in U937 cells. U937 cells were treated with 1.5 μM SEM-Mb for indicated time periods. $[Ca^{2+}]i$ was quantified by a fluorescence plate reader after loading the cells with a calcium indicator (Fluo-4 AM).

Results were shown as fold-increase in fluorescence intensity compared with the control group. (C) Effect of BAPTA-AM, NAC, and GLX351322 on SEM-Mb-induced an increase in $[Ca^{2+}]i$ (mean±SD, *P<0.05; NS, no statistically significant). (D) Effect of BAPTA-AM, NAC, ML-171, and GLX351322 on SEM-Mb-induced ROS generation (mean±SD, *P<0.05; NS, no statistically significant). (E) SEM-Mb induced mitochondrial ROS generation in U937 cells. Mitochondrial ROS levels were measured using mitochondrial superoxide probe MitoSOX Red. The data represent the mean±SD. Effect of Mito-TEMPO, BAPTA-AM, NAC, and GLX351322 on SEM-Mb-induced (F) the production of mitochondrial ROS, (G) $\Delta\Psi m$ loss, and (H) cell death (mean±SD, *P<0.05).

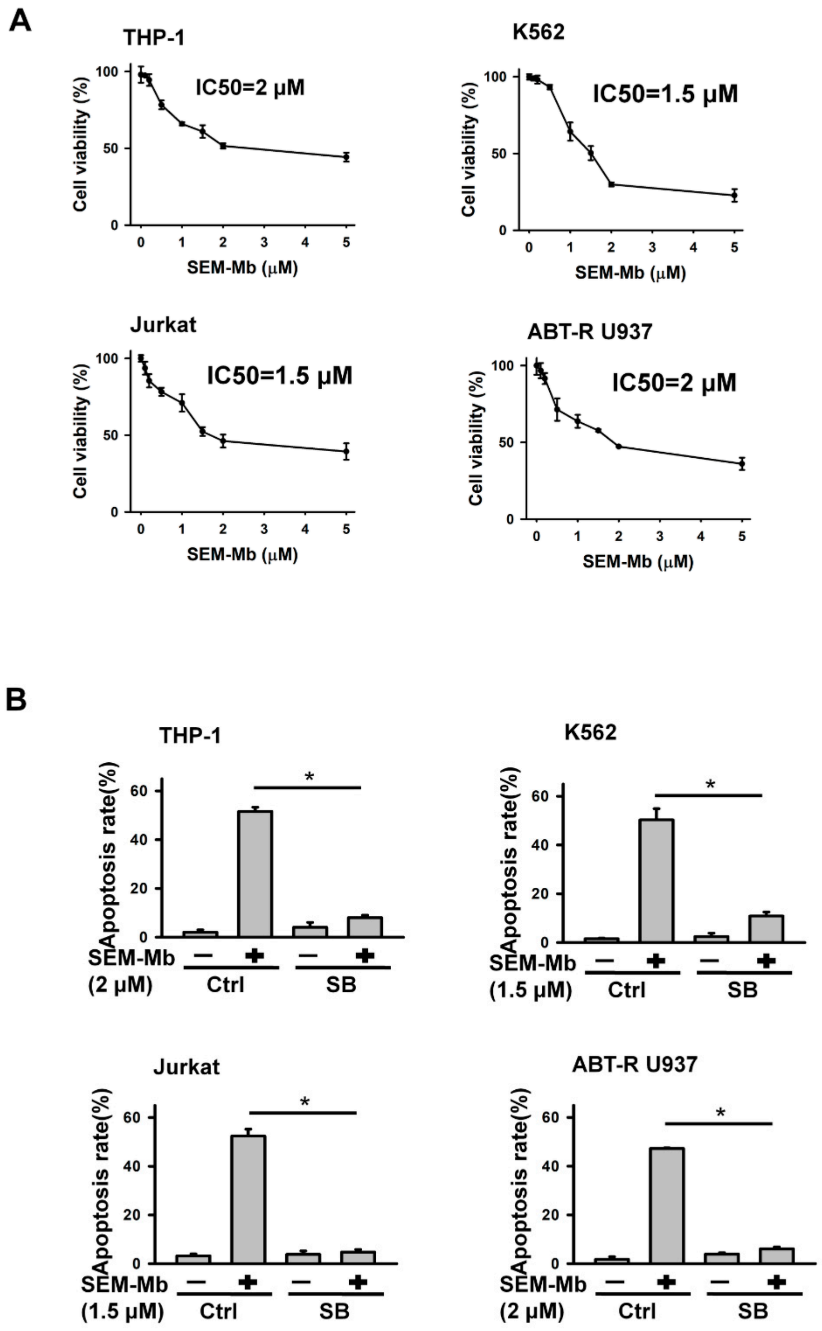
A
THP-1
IC50=2 μM
Cell viability (%)
SEM-Mb (μM)
K562
IC50=1.5 μM
Cell viability (%)
SEM-Mb (μM)
Jurkat
IC50=1.5 μM
Cell viability (%)
SEM-Mb (μM)
ABT-R U937
IC50=2 μM
Cell viability (%)
SEM-Mb (μM)
B
THP-1
Apoptosis rate(%)
SEM-Mb
(2 μM)
Ctrl
SB
K562
Apoptosis rate(%)
SEM-Mb
(1.5 μM)
Ctrl
SB
Jurkat
Apoptosis rate(%)
SEM-Mb
(1.5 μM)
Ctrl
SB
ABT-R U937
Apoptosis rate(%)
SEM-Mb
(2 μM)
Ctrl
SB

C

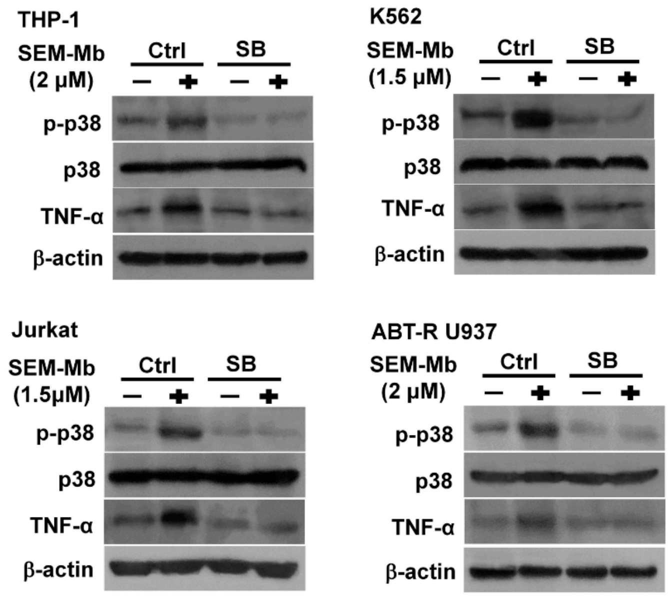

Figure S2. SEM-Mb induced death of THP-1, K562, Jurkat, and ABT-199-resistant U937 cells via p38 MAPK-mediated TNF-α expression. THP-1, K562, Jurkat, and ABT-199-resistant U937 (ABT-R U937) cells were treated with indicated SEM-Mb concentrations for 24 h. (A) SEM-Mb induced cell death in a concentration-dependent manner. THP-1, K562, Jurkat, and ABT-R U937 cells were incubated with varying concentrations of SEM-Mb for 24 h. Cell viability was determined by MTT assay. Results are expressed as the percentage of cell survival relative to the control. Each value is the mean±SD of triplicate determinations. (B) Effect of SB202190 on SEM-Mb-induced apoptosis in THP-1, K562, Jurkat, and ABT-R U937 cells. The cells were pretreated with 10 μM SB202190 for 1 h, and then incubated with indicated SEM-Mb concentrations for 24 h. Apoptosis was assessed in triplicate by annexin V-propidium iodide double staining followed by flow cytometry, and percentage apoptosis is shown as percentage of annexin V-positive cells (mean±SD, $^{*}P < 0.05$). (C) Effect of SB202190 on SEM-Mb-induced TNF-α expression in THP-1, K562, Jurkat, and ABT-R U937 cells.

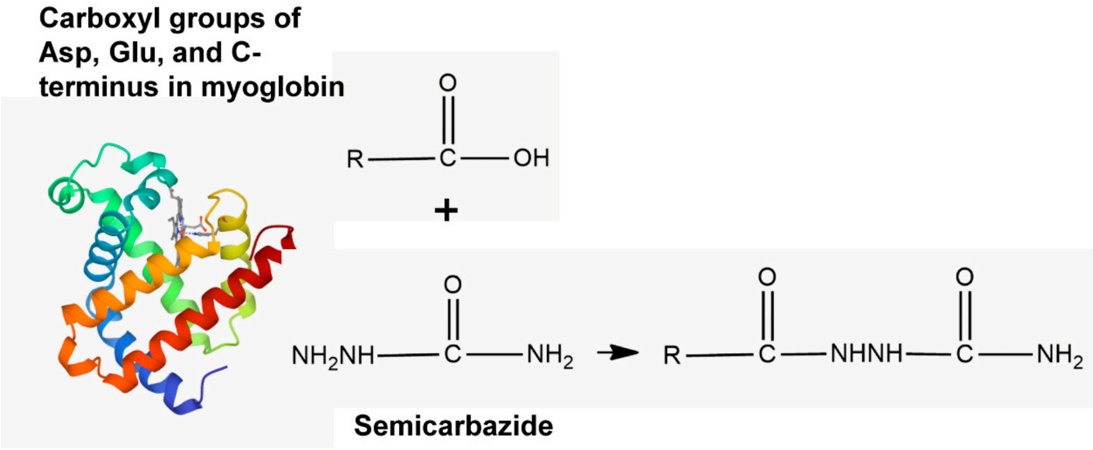

Figure S3. Schematic showing the modification of the carboxyl group in Mb with semicarbazide. Carboxyl groups in Mb were modified with semicarbazide according to the procedure described in [25].

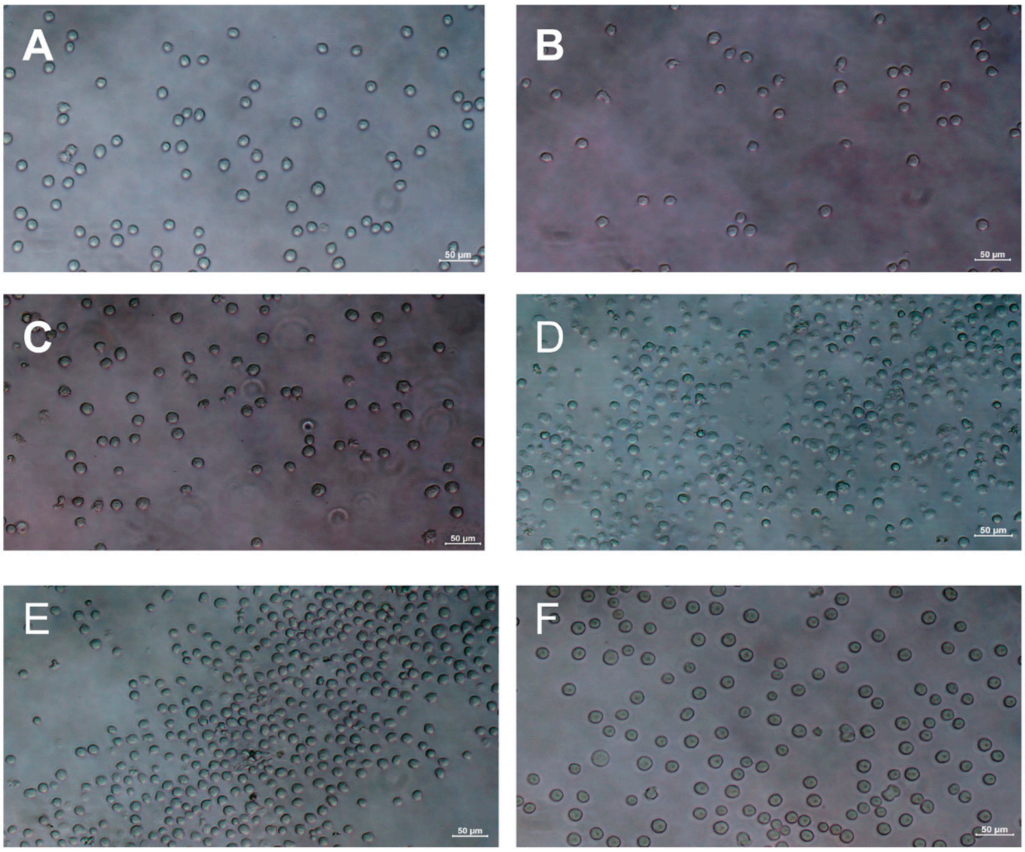

Figure S4. The morphologies of (A) U937, (B) ABT-199-resistant U937 cells, (C) HL-60, (D) THP-1, (E) Jurkat, and (F) K562 cells.
